# The American Academy of Medicine

**Published:** 1878-02

**Authors:** 


### THE AMERICAN ACADEMY OF MEDICINE.

PITTSBURGH, PA., Dec. 31, 1877.

*To the Editor of the Medical Journal and Examiner:*

In the December number of your most valuable journal appears an article from the pen of Dr. M. W. Wood, U. S. A., on a "Proposed Plan for a Medical Reform."

The plan is a good one, but it is not my object to examine it in detail at this time. My object is to call attention to the American Academy of Medicine, by asking you to publish the following articles from its constitution, also the notice as to manner of procuring further information in regard to the organization.

Very truly yours,

R. STANSBURY SUTTON.

### ARTICLE I.

*Name of the Society.*

This Association shall be known as the American Academy of Medicine.

### ARTICLE II.

*Objects of the Society.*

The general objects of the Society shall be the extension of the bounds of medical science; the elevation of the profession, etc., etc.

The special objects of the Society shall be:

1st. To encourage young men to pursue regular courses of study in classical, scientific and literary schools of the highest grade, before entering upon the study of medicine.

2d. To bring together into closer relations, the alumni of such institutions, and to perpetuate their names in the history of the profession.

3d. To secure recognition abroad by medical societies of the highest standing.

### Article III.

*Members of the Society.*

Sec. I. The Society shall consist of active, corresponding and honorary members.

Sec. II. The active members of the Society shall be alumni of respectable institutions of learning, classical, scientific and medical. They shall have conferred upon them by such institutions:

1st. The degree of Bachelor of Arts, after a regular course of study extending through a period of time not less than five years.

2d. The degree of Master of Arts in accordance with the usages of such schools; and,

3d. The degree of Doctor of Medicine after a course of study not less than three years, under the direction and instruction of preceptors and professors.

Sec. III. In the case of those who have pursued regular courses of study in foreign universities in which the degree of Doctor of Medicine covers the ground of a thorough academic course, no other degree shall be required.

Sec. IV. Every applicant for membership shall have an experience of three years in the practice of medicine in one or more of its recognized departments, and shall have a good moral character.

### Article IV.

*The Officers of the Society.*

Sec. I. The officers of the Society shall be a president, four vice-presidents, a recording secretary, a corresponding secretary, and a treasurer, who shall be elected annually, etc., etc.

To secure in the future a higher standard of qualifications than is herein mentioned, and to encourage all institutions of learning in our country conferring degrees, to raise their standard to a level with that of European institutions, the following article is presented for special consideration :

